# Biomedicines in Longevity and Aging the Quest to Resist Biological Decline

**DOI:** 10.37796/2211-8039.1433

**Published:** 2024-03-01

**Authors:** Raymond D. Palmer

**Affiliations:** Spartan Therapeutics Dubai, United Arab Emirates

**Keywords:** Telomere attrition, Telomerase, Immune senescence, Mitochondrial dysfunction, Glycation, mTOR

## Abstract

Aging is considered part of the natural process of life, however in recent years medical literature has started to show that specific facets of aging are beginning to be understood and those factors may even be considered preventable with various measures.

Aging is also considered the number one cause of poor quality of life, disease, disability, and death, so the importance of understanding the aging process and how to control certain aspects of it cannot be underestimated when age related suffering is factored in. The causes of aging are now becoming well understood, and in recent years many therapies have already become available to the public to attenuate specific corridors of aging. The heterogeneity of the aging process and the biological drivers involved is examined here in parallel with various compounds and therapies to combat biological decline.

The benefits for governments in keeping their populations healthy and vibrant are vast, and at the same time offer a great incentive to invest into newly emerging technologies that may prevent the onset of preventable disease.

Whilst this paper only discusses nine pathways to the aging process, many more exist.

Key summary points Aim: Can facets of the aging process be mitigated with new technologies?

Findings: This review finds data which indicates that various aspects of aging can be attenuated.

Message: Aging is a multi-pronged challenge that must be approached from all biological angles.

## 1. Introduction

Aging may be considered a side effect from years of biological insults, lifestyle and inherited genetic traits, along with biological limitations such as stem cell exhaustion, telomere attrition, epigenetic drift, immune senescence, oxidative stress, genomic replicative errors, arterial calcification, osteoporosis, neurological decline and much more.

Heterochronic parabiosis was the first experiment that demonstrated that a restoration of function in some tissues could transpire in vivo which appeared to ameliorate/counteract aging, where older mice would appear to become more youthful again simply by sharing circulatory systems and receiving the blood of younger mice [1,2].

Aging could be associated with a gradual loss of homeostatic mechanisms that maintain the structure and function of adult tissues, particularly the nervous system. Cellular insults such as ultraviolet light, lack of essential coenzymes such as nicotinamide adenine dinucleotide (NAD) or reduced levels of the body's antioxidants such as glutathione [3], the three super oxide dismutases [4,5] loss of mitochondrial function unable to power the cell efficiently [6], including mutations in the genetic code itself [7] all eventually come back to plague human biology in the form of age-related pathologies. So, could restoring those cellular factors back to youthful levels prevent the onset of the pathologies they would otherwise generate?

One of the hallmarks of cellular aging is an accumulation of damaged macromolecules such as deoxyribose nucleic acid (DNA), proteins, and lipids [8]. These become chemically modified by reactive molecules such as free radicals that are generated during normal cellular metabolism and increase with age due to decreasing levels of the body's natural anti-oxidants [9].

Even epigenetic dysfunction or genomic instability due to molecular events resulting in tumourigenesis from carcinogenic environmental pathways can deliver a disease or early mortality. Inadequate lifestyle choices speed up the effect of age-related pathologies such as genomic instability and cellular dysfunction that induce the onset of age-related disease [10].

**Fig. 1:**
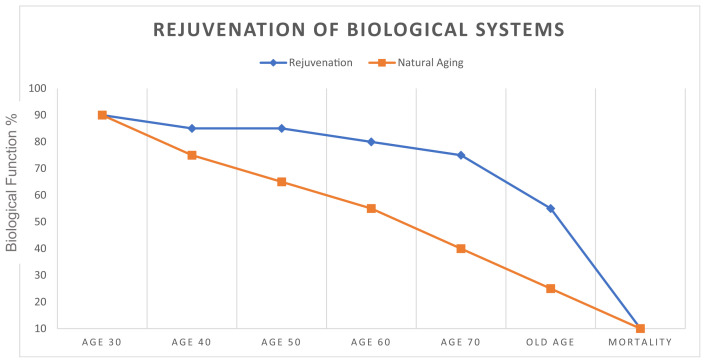
The graphical abstract depicts a theoretical the natural decline caused by aging, and the possible extension of health span when rejuvenation technologies such as the ones discussed herein are implemented.

The goal of this paper is to demonstrate that interventions exist to resist biological decline as shown below in Fig. 1.

Multiple drivers certainly exist in the aging process, and it appears that no single molecule will ever be able to fully control aging due to the heterogeneity and complexity of aging itself; it is much more conceivable that because there are multiple drivers of aging that this will be confronted with a multifaceted approach in order to deliver humanity into an extended health span with the possibility of an extended life span.

Some of the most well-studied gene families shown to influence longevity, either through antiaging or pro-aging effects, are the insulin receptor signalling pathway [11,12], FOXO family of transcription factors [13–15], Sirtuins [16,17], and mechanistic target of rapamycin (mTOR) [18,19].

This paper will discuss nine known drivers of the aging process and how those pathways may be controlled or influenced with non-invasive techniques to possibly ward off age related decline. Various drivers of aging may overlap; however, for the purposes of this review they have been separated and treated as their own single condition, so the rejuvenation is clear for the purposes of this discussion.

## 2. Discussion

Pathways for Biological Descent.

1. Mitochondrial Dysfunction
2. Telomere Attrition – Cellular Divisional Limits
3. Immune Senescence
4. Diminishing Enzyme and Antioxidant Levels
5. Epigenetic Drift – The Loss of Regenerative Genes
6. Glycation – Advanced Glycation End Products
7. Overexpression of Rampant mTOR
8. Inflammation
9. The FOXO Pathway

### 2.1. Mitochondrial dysfunction (MD)

MD has been implicated as a possible precursor to multiple pathologies [6]. During life, the energy required for metabolic functions is supplied by a small organelle called mitochondria located in the cytoplasm of the eukaryotic cell. This energy comes as a molecule known as adenosine triphosphate (ATP) [20]. As humans age, the energy production from mitochondria decreases making exercise and other functions for the cell harder. This presents a downward cascade into biological decline as healthy cell function is inhibited resulting in the possible onset of age-related deterioration and disease [21]. Many advances have been made in mitochondrial knowledge and medicine with various compounds to prevent MD, with one of those compounds called pyrroloquinoline quinone (PQQ) also known as methoxatin which has been shown to increase mitogenesis by activating mitochondrial biogenesis genes [22]. PQQ is found in egg yolks and milk which may indicate PQQ's immediate importance for embryonic or newborn development [23]. Furthermore, other contenders exist in mitochondrial health such as the endogenous metabolite α-ketoglutarate (AKG) which is found inside the mitochondria and functions as part of The Krebs Cycle [24]. AKG was found to extend life in model organisms by 10.5 %, and increased health-span by 40 %. The interpretation of this data shows that AKG extends the time that the model organisms exhibited a younger phenotype more than it extended life. AKG (2 %) also promoted skeletal muscle hypertrophy in mice, indicating that it may also resist aging phenotypes such as sarcopenia [25].

The mitochondrium is susceptible to excess free radical damage due to its ability to create abundant energy for the cell [26], however this free radical damage may be mitigated by PQQ as it is a very capable antioxidant [27]. A more powerful approach is the recently developed suppressor of site IQ Electron Leak (S1QEL1.719) which acts on site I_Q_ at complex 1 inside the mitochondria. S1QEL1.719 is able to prevent the leak of electrons from the electron transport chain without interfering with normal redox signalling [28]. The genes that activate mitochondrial biogenesis also assist in sugar metabolism, cell division and function, standard body weight and normal fat metabolism [29]. MD and the resulting cascade into cellular dysfunction which leads to severe pathophysiologies may now be manageable with easily absorbed compounds such as S1QEL1.719, PQQ or AKG. The data on how profound molecules such as S1QEL1.719 will be for *Homo sapiens* remains in its infancy, however the technology to upregulate mitogenesis or ATP production whilst reducing oxidative damage is now promising.

### 2.2. Telomere attrition

Cell division causes the telomeric caps of chromosomes to shorten during replication [30]. It has been demonstrated that the shorter telomeres become the more cellular dysfunction may ensue, so an inference could be drawn that telomere length is also an indicator for cellular health [31]. Moreover, if telomeres become excessively short cells can no longer divide, at which point cells should enter apoptosis. However some cells may not follow apoptosis and may enter a state known as senescence [32]. The divisional limits on cells from telomere length is known as the Hayflick limit [33]. Telomere attrition was first discovered by Nobel Laureates Elizabeth Blackburn, Jack Szostak and Carol Greider [34]. An enzyme known as telomerase can prevent further loss of telomeres [35]. Telomerase activity was found to be higher in peripheral blood mononuclear cells and was shown to decline with the onset of aging [36,37], and supplementation of telomerase via oral or intranasal would not be successful due to stomach acids, immunogenicity (adaptive and innate), microbiome breakdown of small molecules in the stomach, and the lack of cell surface and nuclear transporters to deliver such a large enzyme into the nucleus. Telomerase remains active in regenerative tissues and in cancer cells, though is downregulated in most of the somatic tissues [38]. This active state of telomerase in cancer cells means that cancer cells remain immortal, as these cells never reach a divisional limit [38]. However, new biomedicines are being developed and peptides have become a front runner for the elongation of telomeres, with Epithalon (also known as epitalon) inducing the expression of telomerase catalytic subunit and associated enzymatic activity [39]. Epithalon is a relatively small tetrapeptide with an alanine > glutamic acid > aspartic acid > glycine (Ala-Glu-Asp-Gly) sequence. Epithalon is also shown to regulate the pineal gland, retina and brain, and also induces neuronal cell differentiation in retinal and periodontal ligament stem cells [39,40]. Furthermore, a polyamine known as spermidine is also another interesting target for the prevention of telomere attrition. In a study where spermidine was administered to promote autophagy in mice, it was also found that spermidine increased telomere length [41]. Other compounds also exist to resist telomere attrition such as ergothioneine, and cycloastragenol which is an aglycone of Astragaloside [42,43]. Side by side comparisons have also been performed for other compounds by Tsoukalas et al that showed the effects of various telomerase activators (from most potent to least potent) being Centella asiatica extract formulation, oleanolic acid, an Astragalus extract formulation, TA-65 containing Astragalus Membranaceus extract and maslinic acid [44] Human trials are warranted for further confirmation on the efficacy of the aforementioned telomerase activators. Studies that only show results in vitro or model organisms should not be considered results that will convey over to humans as such molecules (if orally administered) must survive the first pass effect and also evade immunogenicity and the microbiome [45]. Currently the easiest way to increase telomere health is exercise [46]. The importance of maintaining cellular populations is paramount, as fewer cells concludes fewer proteins for tissues or enzymatic rejuvenation functions. Much more research is necessary in the fight against telomere attrition.

### 2.3. Immune senescence

T-cell populations generated via the thymus gland begin to decline during age which decreases immune system function to fight infection [47]. As the thymus gland shrinks throughout age, thymopoiesis takes hold resulting in ever decreasing amounts of T-cells leading to increased infection and severity in older people [48]. Regenerating the thymus gland in older people is now a viable target for research. This condition of a weakened immune system is often referred to as immune senescence [49]. Thymus regeneration technologies to boost T-cell abundance now exists. The thymus regeneration, immunerestoration and insulin mitigation (TRIMM) trial which targeted thymus rejuvenation by Fahy et al., were able to regenerate thymus size and function, but were also able to reverse methylation markers to a more youthful state [50]. The enrolled subjects were treated with recombinant human growth hormone plus dehydroepiandrosterone and metformin to prevent hyperinsulinemia and the diabetogenic side effects of growth hormone administration. Effects of treatment were measured on immune cells, cytokines, and epigenetic clocks to indicate biological age. Immunological measures improved in the treated participants and methylation markers were clearly shown to have reverted to a more youthful expression pattern [50]. The TRIMM trial was the first of its kind that not only restored youthful function to an organ of the human body (thymus), but the combination of already available, well-established and understood biomedicines also restored DNA methylation expression patterns to a more youthful configuration. In the post era of Covid 19, any therapy that can restore immune function in the elderly may be of paramount importance. The TRIMM trial was also conducted in humans with further research currently underway. In addition to thymus involution, immunosenescence is also driven by decreasing levels of telomerase which interfere with the replicative ability of memory T cells. Telomerase activity can be upregulated by CD28 costimulatory signalling, however Plunkett et al found that reduced expression of telomerase activity in highly differentiated CD8(+)CD28(−)CD27(−) T cells depicted their drift toward their replicative end stage [51]. This data shows the limited ability of memory CD8(+) T cells to perform continuous proliferation which is a factor in immunosenesence.

### 2.4. Decreasing antioxidant levels

The human body contains (on average) 37 trillion cells [52]. Each cell contains a complex network of organelles that perform functions which are imperative for (most notably) cellular energy, maintenance, differentiation and replication of genetic information and genomic stability. These trillions of cells also produce free radicals whilst carrying out a myriad of essential functions. NAD is a primary regulator in cellular and genomic health, and therefore could be considered a master regulator in aging [53]. NAD is critical in the production of adenosine triphosphate for cellular energy production. NAD is also paramount in DNA repair machinery such as Poly (ADP-ribose) polymerase 1 (PARP1) [54]. PARP1 is a nuclear enzyme found in the DNA repair pathway. The effect of the PARP1 mechanism is to repair single or double strand breaks from being copied or joined incorrectly. However, PARP1 requires sufficient levels of NAD to function efficiently, and when NAD levels are low PARP1 can no longer perform its function which may result in missense variants being replicated that may lead to cancer. When NAD levels decline, cells cannot function aptly, and this may be a potential birthplace of where some disease may stem from. Glucose metabolism, cardiovascular function, stem cell health, neural function are all regulated by NAD levels [31]. Rajman et al. have effectively demonstrated how important NAD is in restoring communication between the cell nucleus and mitochondria. These declining NAD levels are easily attenuated during age with over-the-counter NAD precursors. NAD trials are now delivering datasets with conditions such as psoriasis and skeletal muscle activity showing promising results along with inducing a transcriptomic signature and suppressed inflammatory cytokines [55,56].

Endogenous antioxidants also diminish throughout age. Notably glutathione, the three super oxide dismutases (SOD1, SOD2 and SOD3) and catalase which could be regarded as the body's primary biomedicines, are all major players in the fight against free radicals and the pathophysiology of aging [2].

Whether this decreasing of the body's antioxidants is a result of the aging process or a driver of the aging process remains to be seen, yet much of these endogenous antioxidants can be restored with simple supplementation. Again, it is observed that a relatively simple approach can restore youthful levels of essential biological mechanisms to ward off the aging process. Methods of delivery such as oral, intranasal, transdermal patches or IV drip are still matters of contention, though the evidence shows that consumers can safely use some oral products to reclaim their ability to fight against free radical damage [32]. Glutathione that is administrated orally is poorly absorbed due to its swift hydrolysis by γ-glutamyltransferase existing in intestinal mucosa, hepatocytes and cholangiocytes [57]. N-acetylcysteine (NAC) has become a well-known precursor in raising glutathione levels, though human trial data on the best dosage and delivery methods are lacking [58]. Super oxide dismutase is found in three distinct regions. SOD1 is cytoplasmic, whilst SOD2 is mitochondrial and SOD3 is extracellular [59]. It should be noted that reactive oxygen species also increase with age creating a disbalance between oxidants and anti-oxidants [60].

### 2.5. Epigenetic drift

The epigenome regulates human gene expression via imprinting centres, methylation, acetylation, CpG islands, histones and more. The epigenome could be considered a regulator of gene expression [61]. Many genes that can cause disease are silenced by the epigenome whilst other genes that deliver a youthful physiology are transcriptionally switched on to produce proteins. As humans’ age the epigenetic machinery begins to drift, lowering expression on genes that are abundant during youth which may exasperate the onset of old age. There are many theories and research papers on why the epigenome drifts with age, and interesting techniques to suppress this phenomena with dietary supplements is fast becoming a contender in the fight to lock the epigenome into the most disease resistant and youthful configuration [62,63].

The epigenetic diet is comprised of foods that appear to restrict or revert methylation patterns to earlier states. The University of Uppsala has demonstrated that the simple ingestion of tea may upregulate specific gene expression due to influence over methylation [64]. Other epigenetic regulators are curcumin, broccoli, kale, cabbage, watercress, and folate. Folate has also been shown as a prominent epigenetic regulator [65]. Foods rich in selenium are also implicated as attenuators of epigenetic drift [66]. This data leads into the conclusion that the epigenome can be influenced with sensible dietary choices. Furthermore, trials that have altered epigenetic drift have now been conducted and clearly show (as with the TRIMM trial discussed earlier) a reversal in epigenetic expression. Controlling such powerful mechanisms inside the nucleus of the cell will no doubt yield great benefit for humanity's quest to stay healthy.

### 2.6. Glycation

The process of non-enzymatic changes to lipids and proteins once exposed to monosaccharides is known as glycation [67]. These monosaccharides interrupt or disrupt various signalling processes so that the cell cannot receive or send signals efficiently. This can lead to improper or total lack of cell function, cell death or cell senescence. Glycation can have a flow on effect onto proteins in different cell types including liver, eyes, heart, and brain. An excessive level of glycation can result in a plethora of pathophysiologies. Sugary food and drinks are absorbed by the body and these monosaccharides then cause reactions known as 'advanced glycation end products (AGEs) [68]. However, the accumulation of AGEs can be controlled with low glycaemic foods [69].

Building muscle requires glucose resulting in the cleaning up and removal of AGEs [70]. Antioxidants are also a defence mechanism against AGEs, and healthy diets rich in fruit and vegetables could be considered the first line of defence. Cheese contains high levels of AGEs and vegetable fats contain fewer AGEs than animal fats. Concentrated fatty dairy foods such as creams and butter can also contain high levels of AGEs [71]. The accumulation of AGEs is greatly affected by the choices of the individual. Many studies are available that demonstrate clear data in humans regarding the deleterious effect that AGEs produce [71]. Diet is clearly the primary damage mechanism from AGEs.

**Mammalian Target of Rapamycin** (mTOR) mTOR inhibitors are a class of drugs designed to block the mTOR signalling and growth pathway. This class of drugs shows great promise with suppressing tumour growth and aging [72]. Metformin and Rapamycin are both common drugs used for the treatment of diabetes; however recent data indicates that they may possess longevity benefits by suppressing the mTOR pathway. mTOR is a kinase which acts upstream of autophagy which degrades and recycles damaged molecules and organelles. mTOR is a protein involved in cell cycle progression and regulation, and therefore drives cells to divide, including cancer. Inhibiting the mTOR pathway with signalling suppressors such as Metformin or Rapamycin will prevent further signalling, which then prevents further cell division, which in turn may prolong the time for healthy cells to reach their divisional limit [73].

Metformin is also a fasting mimetic which assists the body with the effects of a fasting diet [74]. This holds additional value due to starvation mechanisms activating under calorie restriction, and furthermore, the body may begin to eliminate various cells from tissues [75]. These cells are usually cells that have become of low level importance or dysfunctional. Other biological products such as stored glucose may also be recycled via the kidney which regulates glucose via gluconeogenesis by taking glucose from the circulation and then using the glomerular filtrate to reabsorb the glucose [76,77]. Metformin is also well tolerated in humans and has been used to deliver anti-diabetic properties since the 1950's [78]. Metformin's major competitor in the antiaging biomedicine race is Rapamycin, which has been shown to extend the lives of short-lived mutant strain mice by up to three-fold [79], and in 2006 was acclaimed to be an anti-aging biomedicine that could be used immediately to slow the onset of aging and disease [80]. Both Metformin and Rapamycin are well tolerated in humans with a good history of relative safety when used properly, however recent data indicates that Rapamycin may induce Alzheimer's beta-amyloid plaques in mice, and further research is required before any definitive conclusion in humans can be reached [81].

#### 2.6.1. Inflammation

Inflammation is a signalling mechanism which evolved to communicate to certain cell types to either fight infection or repair injury. This mechanistic pathway utilises proinflammatory cytokines which transiently assist repair mechanisms [82]. Once repair function has transpired pro-inflammatory cytokines disappear until another infection or injury signals to them. However, it has been observed that during aging these proinflammatory cytokines begin to linger in the blood for extended periods which interfere with numerous healthy tissue functions. Pro-inflammatory cytokines have been connected to blood vessel damage, depression and even arthritis by interfering with stem cell pathways and it should be noted that cytokines are part of the senescence-associated secretory phenotype (SASP) found across all cell types including postmitotic cells [83,84].

Improper diet is now thought to be a major cause of extended inflammation with various foods delivering excessive amounts of Omega 6 fatty acids. Small amounts of Omega 6 have not been shown to be harmful, however the high levels of Omega 6 in certain food products may be harmful to those that are at high risk of inflammation [85]. Furthermore, it should be noted that AGEs are also believed to be a driver of prolonged inflammation. The evidence again points to inflammation as being partly a manmade pathology which may now be manageable with informed lifestyle choices [86].

Specific types of gut bacteria have also been found to be inflammatory in various murine studies [87]. Older mice have increased pathologic diversity, but most notably stiffness in the arteries. The offending bacteria known as Proteobacteria created large amounts of trimethylamine N-oxide (TMAO) which inflicts damage to arterial walls and causes oxidative stress [88]. Inhibiting TMAO appears feasible according to a paper published by Chen & Zhang 2016 [89]. Chen and Zhang demonstrated that supplementation with resveratrol lowered TMAO significantly. Further research is required to determine whether resveratrol will have the same effects in human subjects. Other questions remain such as what other factors can cause elevated levels of TMAO and of note, high protein or high fat diets were also contributors to raising TMAO levels.

Much of the literature for extended bouts of inflammation point to the precursor being lifestyle and dietary choices. Therefore, the case can be argued that simple changes to lifestyle and diet can significantly affect not only the rate of inflammation but also how severe the effects of inflammation can be. However, this discussion should point out that inflammation is beneficial when it is transient for infections or injury repair, however prolonged inflammation appears to be a driver in age related decline.

### 2.7. The forkhead box O (FOXO)

The FOXO family of genes in mammals consist of FOXO1, FOXO3, FOXO4, and FOXO6 which are implicated downstream in critical cellular functions such as stress resistance, cell cycle arrest, apoptosis and metabolism [90]. As humans age the ability of this family of transcription factors becomes diminished resulting in an inability to translate new protein to regenerate tissues. Damaged tissues may result from prolonged environmental insults such as ultraviolet light, free radical stress, toxins, or poor lifestyle choices. FOXO could be regarded as a group of heterogenous family of transcription factors that controls many other downstream genes responsible for regeneration. FOXO activation has been able to extend the lives of ‘c elegans’ by 100 %. It could be reasoned that the genetic systems for longevity are similar for humans as they are in worms [91]. Kenyon's research indicates that when an organism undergoes stress such as rigorous exercise or calorie restriction, the body signals the FOXO genes to activate protector genes to turn on and initiate longevity and repair pathways. Inversely when an organism is not stressed by factors such as exercise or calorie restriction and an abundant supply of food is available, Insulin Growth Factor 1 (IGF-1) appears to turn down the FOXO genes placing an organism into an elevated state of aging. Arguably FOXO can be highly influenced by external factors that most of the population can implement easily such as fasting, exercise and even supplementation with Metformin. Metformin inhibits the signalling cascade that tells the FOXO gene family that food is in abundance. Metformin can also assist with a greater calorie restriction effect as it has been shown to be a fasting mimetic [77]. The FOXO family of genes can be influenced easily by those willing to upregulate this pathway. FOXO appears to be an easy target to manipulate for tissue rejuvenation by well-established mechanisms in humans such as fasting and exercise [91].

## 3. Conclusion

Multiple pathways clearly exist in human aging, and as those aging corridors are becoming better understood, innovative techniques or therapies are also being found or designed to overcome them. This paper does not cover every facet of aging or an exhaustive list of the biomedicines to overcome the drivers of aging, though it does find that various biomedicines now exist to help regulate facets of the aging process.

The literature reviewed in this paper clearly finds a trend that science may be starting to influence how humanity may age. The biosciences are now delivering not only insights into the major causes of aging, but also how to attenuate some of those aging pathways to fend off biological decline to extend health-span and lifespan.

This discussion simply demonstrates that novel biomedicines and treatments already exist to attenuate some of the biological drivers of aging. The benefits for civilization to go beyond a good healthy diet and exercise, but to actively resist facets of aging with therapies such as the ones discussed here are vast. Keeping society out of hospitals, delivering a longer period for our species to be fit and healthy and preventing poor quality of life or disease is no longer a futuristic vision, but a vision that is close to humanity's grasp. Not only can considerable sums of government healthcare spending be saved by ensuring society is kept healthy, but vast human wealth can be generated by keeping the general population fit, vibrant and able to contribute.

## Data Availability

Yes.

## Abbreviations

AGEs: Advanced Glycation End Products
AKG: α-ketoglutarate
ATP: Adenosine Triphosphate
DNA: Deoxyribonucleic Acid
FOXO: Forkhead Box
IGF1: Insulin Growth Factor 1
mTOR: Mammalian Target of Rapamycin
NAD: Nicotinamide Adenine Dinucleotide
NMN: Nicotinamide Mononucleotide
PARP1: Poly (ADP-ribose) Polymerase 1
PQQ: Pyrroloquinoline Quinone
TMAO: Trimethylamine N-Oxide
TRIMM: Thymus Regeneration, Immune-Restoration, And Insulin Mitigation
